# Data and debriefing observations on healthcare simulation to prepare for the COVID-19 pandemic

**DOI:** 10.1016/j.dib.2020.106028

**Published:** 2020-07-15

**Authors:** MH Andreae, A Dudak, V Cherian, P Dhar, PG Dalal, W Po, M Pilipovic, B Shah, W Hazard, DL Rodgers, EH Sinz

**Affiliations:** a Department of Anesthesiology and Perioperative Medicine, Penn State Health Milton S. Hershey Medical Center, Hershey, PA 17033, United States; b Medical Simulation Center, Penn State Health Milton S. Hershey Medical Center, Hershey, PA, United States

**Keywords:** Simulation, Adult critical care, Perioperative care, Acute respiratory syndrome coronavirus, Personal protective equipment, Crisis Resource Management, System Integration, Anesthesiology

## Abstract

We report on data and debriefing observations in the context of an immersive simulation conducted to (a) train clinicians and (b) test new protocols and kits, developed in table-top exercises without prior clinical experience to fit anticipated clinical encounters in the setting of the rapidly expanding COVID-19 pandemic. We simulated scenarios with particular relevance for anesthesiology, perioperative and critical care, including (1) cardiac arrest, (2) emergency airway management, (3) tele-instruction for remote guidance and supervision, and (4) transporting an intubated patient.

Using a grounded theory approach, three authors (MHA, DLR, EHS) developed emergent themes. First alone and then together, we sought consensus in uncovering overarching themes and constructs from the debriefings. We thus performed an informal qualitative thematic analysis based in a critical realist epistemological position - the understanding that our findings, while real, are affected by situational variables and the observer's perspective[1,2]. We compared data from videos and triangulated the data by member checking. All participants and course instructors volunteered to participate in this educational project and contributed as co-authors to this manuscript.

During debriefing, we applied crisis resource management concepts including situation awareness, prioritization of tasks, and clear communication practices, conducting the debriefing with emphasis on current TeamStepps 2.0 terminology and concepts. [3,4] In addition, we re-evaluated formerly familiar processes, as shortcomings of protocols, kits, and interdisciplinary cooperation became apparent. The data provide detailed observations on how immersive simulation and debriefing among peers mitigated the unfamiliarity of individual clinicians and the organization at large with the demands of an unprecedented healthcare crisis. We also observed and report on the anxiety caused by resource constraints, risk to clinicians in the face of limited personal equipment, and the overall uncertainty surrounding COVID-19.

We began to summarize, interpret, critique, and discuss our data and debriefing observations in a rapid co-publication in the Journal of Clinical Anesthesia. [Healthcare Simulation to Prepare for the COVID-19 Pandemic][5]

Specifications table

**Table:** 

Subject	Anesthesiology and Pain Medicine
Specific subject area	Immersive healthcare simulation in perioperative medicine for process improvement and pandemic preparedness
Type of data	Tables
How data were acquired	We converted a previously scheduled MOCA (Maintenance of Certification in Anesthesiology) simulation course [6,7] in our American Society of Anesthesiologists Endorsed simulation program to train internal clinicians in scenarios related to COVID-19 [8,9] and to vet Covid-19 protocols developed in round table discussion by experts, who however had no prior exposure to patients suspected or affected by COVID-19 in the evolving pandemic. Using a grounded theory approach, three authors (MHA, DLR, EHS) developed emergent themes in an informal qualitative thematic analysis[1,2].
Data format	Analyzed
Parameters for data collection	Case Scenarios Simulated Anticipated COVID-19 Clinical Encounters
Description of data collection	Immersive healthcare simulation with seven clinical experts was conducted on four anticipated airborne contagious disease scenarios and the authors analyzed the ensuing debriefing and the case videos.
Data source location	Institution: Medical Simulation Center, Penn State Health Milton S. Hershey Medical Center City/Town/Region: Hershey, Pennsylvania Country: USA
Data accessibility	With the article
Related research article	Co-Publication[5]: Andreae MH, Dudak A, Cherian V, et al. Healthcare simulation to prepare for the COVID-19 pandemic [published online ahead of print, 2020 May 27]. J Clin Anesth. 2020;66:109,928. doi:10.1016/j.jclinane.2020.109928

## 1 Value of the data

• Immersive healthcare simulation employing anticipated clinical encounters may be useful to test COVID-19 [8,10,11] and other airborne contagious disease hospital protocols, developed with limited clinical experience, to detect shortcoming before such gaps become apparent in clinical care and threaten patient or provider safety[12], [13], [14].
• Our report is geared towards other simulation professionals[6,15,16], especially in anesthesiology[17], critical care, and perioperative medicine, who want to leverage immersive simulation to vet their airborne precaution care protocols before the arrival of an epidemic. [12], [13], [14]
• The purpose of our detailed simulation protocols (Table 2) is allow replication of typical airborne contagious disease simulation scenarios and the summary of the emergent themes and key learning points allow others to anticipate, contrast, and triangulate simulation debriefings with participants.
• Given the limited recent experience of healthcare providers in industrialized countries with airborne contagious disease, simulation fill a void not only to train providers in anticipated scenarios, but to test protocols developed in table top exercises without prior clinical exposure, and to augment the organizational response, by improving interdisciplinary coordination[13,14,18].

## 2 Data description

### Table 1

Table 1 tabulates the four simulation scenarios representing anticipated clinical encounters with COVID-19 patients, which we simulated at Penn State Health Milton S. Hershey Medical Center, Hershey, Pennsylvania, USA in March 2020, prior to admitting any COVID-19 patient, with a view to training our providers and testing our COVID-19 protocols in realistic simulation scenarios prior to the arrival of COVID-19 cases at our institution. Case number and Title are in the first and second column on the left, respectively. The Scenario presented to the participant is sketched in the next column and the Central Themes and Key Lessons elicited during our debriefings in the column can be found in the column on the right.

**Table 1:** Case descriptions.

Case	Title	Scenario	Central Theme/Key Lessons
1	Cardiac Arrest for Patient with Possible Communicable Airborne Disease	The team is called to the emergency department to assist with a patient in respiratory distress. During evaluation, patient experiences respiratory arrest and cardiovascular collapse. Goal – Participants will adapt Advanced Cardiovascular Life Support (ACLS) algorithms for a possible COVID-19 patient in cardiac arrest. Focus is on situation awareness during the first minutes of the event, with emphasis on teamwork to provide CPR, defibrillation, airway management, and the first epinephrine administration with modifications for a COVID-19 positive situation.	The central theme is the concept of "Protected Code Blue" where team member safety is emphasized. Procedures are altered to protect the resuscitation team in the context of airborne transmission. Key lessons Huddle outside and establish clear communications to contain airborne contamination. Clinician protection is still a priority even in emergency events. Airborne disease may drive changes in the algorithm such as earlier advanced airway placement and stopping CPR for airway placement. Minimize equipment and clinicians entering the room to reduce contamination and exposure. Integrate procedures across professions or disciplines and ensure scope of practice fits new pandemic practice patterns.
2	Emergency Airway Management for Patient with Contagious Respiratory Disease	Participants are called to assist with the airway management of a known COVID-19 patient in acute respiratory distress to facilitate intubation. The scenario unfolds into an anaphylaxis with a difficult airway. Goal – Participants will modify their approach to securing an emergency airway due to respiratory failure in a positive COVID-19 patient with difficult airway due to anaphylaxis. Focus Is on planning and preparation to provide initial oxygenation and ventilation, management of anaphylaxis, and intubation after respiratory failure.	The central theme is provider safety and containment of airborne transmission during airway management of a COVID-19 patient. Key lessons Airway instrumentation and mask ventilation expose clinicians to virus aerosolization. Dedicated airway kits can be optimized for COVID-19 patients. Airborne disease may lead to different airway tools and management options. Limited tools and increased risk may necessitate faster progression to emergency surgical airway.
3	Transport of a Patient with Contagious Airborne Disease	A known COVID-19 patient needs an in-hospital transport from the ICU to the OR. The patient is intubated on high PEEP and FiO2. He is on multiple infusions including pressors to maintain blood pressure, sedatives, and epoprostenol to improve V/Q mismatch. Participants must prepare the patient for transport and move the patient from the room and down the hall. Goal – Participants will prepare for and transport a COVID-19 positive patient from the Intensive Care Unit to the Operating Room for emergency surgery taking necessary actions to limit virus exposure during the transport while protecting the patient from acute deterioration. Focus is on establishing clear roles for multiprofessional team members and taking actions to reduce potential of virus spread during the transport.	The central theme is on team coordination, communication with hospital entities and adherence to protocol to contain viral spread. Key Lessons Interdisciplinary discussion to evaluate the need for transfer versus performing the procedure in patient room. Limit infusion pumps and other equipment and cover all equipment to reduce contamination during transport. If not already intubated, consider intubation prior to transport to avoid bag mask ventilation and exposure of OR personnel and bystanders. Coordination between “clean” and “contaminated” personnel is paramount.
4	Tele-instruction for Remote Procedural Guidance and Supervision	The participants are tasked to place a chest-tube, but there is no provider available who has experience in placing a chest tube. A provider with experience in chest tube placement remotely directs the bedside clinician on performing the procedure using a Tele-ICU unit or other similar two way- audio/visual system. Goal – Remote team leader will provide instruction and coaching to team members using a two-way audio/visual link (telehealth) to instruct bedside participant how to perform chest tube placement in a patient with a tension pneumothorax that has been temporized with needle thoracotomy. Focus in on clear communication for skills coaching using appropriate telehealth communication practices and techniques to assist the bedside clinician in performing the procedure under remote direction.	The central theme is communication and supervision via remote telecommunication to perform a life-saving procedure. Key lessons If time permits, a dry run or rehearsal improves communication and shared mental modeling for the procedure. Align camera or patient, moving the more mobile of the two, to create better remote viewing point. Remote coach maintains a composed demeanor to induce corresponding calm in the clinician at the bedside. Clear, specific instructions delivered in a calm voice while directing and coaching the bedside clinician enhance success.

**Abbreviations:** ICU: intensive care unit, OR: operating room, PEEP: positive end expiratory pressure, FiO2: fraction of inspired oxygen, V/Q: ventilation/perfusion (lung function).

### Table 2

Table 2 presents additional emerging themes observed during the debriefing and simulation cases conceived, subsequent to our debriefing of the initial four scenarios simulated representing anticipated clinical encounters with COVID-19 patients. These additional scenarios are still awaiting simulation. Cases are numbered in the first column on the left. A succinct Title summarizes the case content. The Scenario presented to the participant is sketched in the next column and the central themes ate elicited in the column Central Theme on the utmost right.

**Table 2:** Additional emergent theses and cases.

Case	Title	Scenario	Central Theme
1	Conflict resolution in triage situations during a global pandemic	A 65 year old female is denied admission despite significant respiratory distress, because the hospital is stretched beyond capacity and has no more beds. The participants have to break the news to the patient and her family. Patient and relatives a visibly upset and then the son brandishes a gun.	The central theme is a candid discussion of triage in a pandemic and the resulting anguish, conflicts and distress, affecting all stakeholders, including the patient, his or her family, the gatekeeper/decision maker, the messenger, who is informing the patient of the decision, the provider in the background and security personnel enforcing the triage decisions.
2	Ventilating more than one patient with one ventilator during COVID-19	The participants are informed that we have fewer ventilators than patients needing support. The group is asked to setup a ventilator to ventilate four manikins. Given the shortage of ventilators, this could increase the number of patients we can save.	The central themes are (1) the technical execution assembling the parts to connect four patients to one ventilator, (2) the trouble-shooting different and changing lung compliance and the resulting complications of this setup, and (3) the perspective of the participants on this desperate intervention.
3	Family discussion about terminal extubation in an elderly COVID-19 patient with a poor prognosis	Participants are asked to discuss goals of care with the family of an elderly COVID-19 patient with a poor prognosis, in the face of an acute shortage of ventilators.	The central theme is the distress of family and providers facing a grim ethical dilemma of resource allocation in the context of a pandemic.

### Table 3

Table 3 offers a detailed description of the enacted four clinical scenarios of anticipated COVID-19 encounters with objectives, equipment and supplies as well as resources needed, a description of the roles, and the sequential development of the scenario to allow for an easy replication of the reported healthcare simulation scenarios.

**Table 3:** Detailed scenario descriptions and case templates.

Case 1: Cardiac arrest for patient with possible communicable airborne disease Case 2: Emergency airway management for patient with contagious respiratory disease Case 3: Transport of a patient with contagious airborne disease Case 4: Tele-instruction for remote procedural guidance and supervision
*Case 1: Cardiac arrest for patient with possible communicable airborne disease*Goal – Participants will adapt Advanced Cardiovascular Life Support (ACLS) algorithms for a possible COVID-19 patient in cardiac arrest.Objectives – Participants will… 1 Employ CRM techniques including role assignments (e.g., team leader, “dirty” and “clean” team members, task assignments) and communication practices that enhance the team's shared mental model (e.g., huddle prior to room entry, closed loop communication). 2 Implement ACLS algorithm treatments including CPR, defibrillation, airway management, and first medication administration. 3 Protect team members’ exposure by using appropriate PPE and limiting clinicians at the bedside. 4 Modify standard ACLS care and team actions to the COVID-19 situation, including: a Securing advanced airway early to limit aerosolization from bag/mask device b Stopping CPR for airway procedures c Using a video laryngoscope instead of direct laryngoscopy Equipment and supplies – • COVID-19 specific Personal Protective Equipment (e.g., mask, face shield, gown, gloves and/or PAPR) • Code/Crash cart with emergency medications • Oxygen delivery devices (mask, nasal cannula) • Advanced airway kit (Note: this simulation used a custom kit assembled specifically for COVID-19 interventions) • Defibrillator • Step stool for CPR provider Simulator and environment – • Manikin capable of advanced airway placement, provision of chest compressions, and production of EKG readable by defibrillator using electrode pads • Manikin on a height adjustable gurney • Vital signs (EKG, NIBP, EtCo2, and SpO2) on display • IV in place, normal saline at KVO rate • Wearing hospital gown • Bedside table for placing equipment • Stethoscope In Scenario actor – • Faculty member in role of bedside clinician to supply relevant patient information. Faculty member is not in full PPE (necessitates direction of team leader to remove her/himself from area) Scenario duration – • Briefing – 3 min • Simulation – 12 min • Debriefing – 15 min Briefing –“Your team has been called to the Emergency Department to assist with a patient as the existing ED physicians and staff are overwhelmed by a sudden surge in patients presenting to the hospital, many with respiratory symptoms.”Simulation Progression –In scenario actor meets team in hallway outside patient room, stating…
• “I am the nurse assigned to this patient. We have a 40-year-old male patient who has presented with fever and cough, since admission he has been complaining of increased respiratory distress.”
Upon questioning, the nurse supplies the following… • There is no remarkable medical, surgical, or social history and no medication or allergies. • He is a sales manager with a 30-year history of one pack a day smoking. • He recently flew back from a company conference attending by over 200 people from around the world. • He does not recall being exposed to anyone who was sick. Patient Presentation – • Patient is in respiratory distress stating he is having a “hard time breathing.” Depending on simulation program and manikin capabilities, patient (manikin) may respond to questions or information can be supplied by in scenario actor. Physical examination – • Patient weight is 98 KG (slightly obese) • Lung sound indicate rales Vital Signs (upon application of monitors) • Heart Rate – 102 • Respiratory Rate – 26 • Blood Pressure – 109/78 • SpO2 – 91% on room air • Temperature – 38.9 C Initial State (3 min) • Patient steadily develops worsening of respiratory distress State 2 – Cardiac Arrest • The patient deteriorates, loses consciousness, and stops breathing. He no longer has a pulse. Monitors show ventricular tachycardia. State 3 – Return of Spontaneous Circulation (ROSC) • After defibrillation (initial and subsequent), advanced airway placement, and first IV epinephrine administration, patient has ROSC and returns to baseline vital signs; however, remains unconscious. Scenario ends. Expected actions – 1 CRM principles used to identify team roles and responsibilities. 2 Appropriate PPE donning prior to room entry. 3 Team leader limits number of people in room (may require expansion of scope of practice to accommodate all tasks). 4 Team leader recognizes bedside clinician without appropriate PPE and removes him/her from space for decontamination. 5 Team leader limits equipment in room (crash cart remains outside room in “clean” area). Essential equipment carried into room. 6 Team leader implement ACLS algorithm with following modification: a Securing advanced airway early to limit aerosolization from bag/mask device b Stopping CPR for airway procedures c Using a video laryngoscope instead of direct laryngoscopy 7 Communication between “dirty” team in room and “clean” team in out-of-room support roles is maintained. Debriefing Guide –The participants discuss their response with the facilitator focusing the discussion on the COVID-19 relevant aspects of the scenario. In particular, the group discusses: • donning and doffing of personal protective equipment given resource constraints and the time required to don PPE, • how many clinicians should enter the room and what roles do they assume, • clear identification of team leader, • what equipment should be brought into the room (with regards to decontamination or destruction afterward), • how to maintain communication between the “dirty” and “clean team members to solicit assistance and additional equipment, • how the code algorithm should be altered in the setting of COVID-19, • what resources need to be activated to bring the patient to the final disposition, ideally a negative pressure room in the intensive care unit.
The scenario may evoke feelings of anxiety and distress in the participants, which may come up in the discussion. Contingent on the familiarity of the participants with each other, it may be challenging to lead a discussion about concerns that touch on personal safety, professional ethics, and professional identity.Distress may be caused by: • prioritizing care in the setting of insufficient hospital or ICU beds, • the delay in providing care (due to the cumbersome process of donning personal protective equipment), • the inability to assist in the code, or • the stress of performing cardio-vascular resuscitation with limited clinicians in the room. Anxiety may be induced by • the uncertainty surrounding COVID-19 and its fatality rate, • confusing and unclear communication by leadership, • the lack of healthcare resources and absent coordinated action to confront the situation, or • the lack of personal protective equipment and resulting concerns for participants health or the health of family members who may be at risk due to immunocompromise, co-morbidities, simply or old age. *Case 2: Emergency Airway Management for Patient with Contagious Respiratory Disease*Goal – Participants will modify their approach to securing an emergency airway due to respiratory failure in a COVID-19 positive patient with difficult airway due to anaphylaxis.Objectives – Participants will… 1 Employ CRM techniques including role assignments (e.g., team leader, “dirty” and “clean” team members, task assignments) and communication practices that enhance the team's shared mental model (e.g., huddle prior to room entry, closed loop communication). 2 Establish an advanced airway in a COVID-19 positive patient in respiratory failure that has been unresponsive to initial medication therapy for anaphylaxis. 3 Protect team members’ exposure by using appropriate PPE and limiting clinicians at the bedside. 4 Modify standard approach to securing an advanced airway for a positive COVID-19 patient. including: a Securing advanced airway early to limit aerosolization from bag/mask device. b Using a video laryngoscope instead of direct laryngoscopy. c Advancing to surgical airway quicker for the CICO patient consistent with the VORTEX approach [24]. Equipment and Supplies – • COVID-19 specific Personal Protective Equipment (e.g., mask, face shield, gown, gloves and/or PAPR) • Code/Crash cart with emergency medications • Oxygen delivery devices (mask, nasal cannula, high flow nasal cannula) • Advanced airway kit (Note: this simulation used a custom kit assembled specifically for COVID-19 interventions) • Surgical airway kit (Note: As a result of this simulation, custom airway kit was modified to include emergency surgical airway supplies) Simulator and Environment – • Manikin capable of advanced airway placement and surgical airway • Pharyngeal edema and tongue swelling activated • Makeup applied to simulate rash on upper chest and neck • Manikin on a height adjustable gurney • Vital signs (EKG, NIBP, EtCo2, and SpO2) on display • IV in place, normal saline at KVO rate • Wearing hospital gown with surgical mask in place • Bedside table for placing equipment • Stethoscope In Scenario Actor – • Faculty member in role of bedside clinician to supply relevant patient information. Faculty member is in full PPE appropriate for a COVID-19 patient. Scenario Duration – • Briefing – 3 min • Simulation – 12 min • Debriefing – 15 min
Briefing –“Your team has been called to an inpatient room for a known COVID-19 positive patient in acute respiratory distress.”Simulation Progression –In scenario actor meets team in hallway outside patient room, stating… • “I am the nurse (or hospitalist) assigned to this patient. We have a 56-year-old female patient who has just received her first treatment with a novel antiviral agent for COVID-19. She started to complain of increased difficulty breathing.” Upon questioning, the nurse supplies the following… • There is no remarkable medical, surgical, or social history and patient is allergic to sulfa medications and penicillin. • She is a laboratory technician, non-smoker, and no drug use history. • She has no recent travel history and no known contacts with COVID-19 confirmed individuals. Her son did recently travel and had been complaining of a low-grade fever since returning. • She has been short of breath, but quickly developed increased respiratory distress over the past 20 min. Patient Presentation – • Patient is in respiratory distress stating she cannot catch her breath. She is having difficultly speaking. Depending on simulation program and manikin capabilities, patient (manikin) may respond to questions or information can be supplied by in scenario actor. Physical examination – • Patient weight is 88 KG. Height is 173 cm. • Lung sounds indicate stridor • Rash note on upper chest and neck Vital Signs • Heart Rate – 99 • Respiratory Rate – 22 • Blood Pressure – 99/78 • SpO2 – 93% on room air • Temperature – 38.9 C Initial State (3 min) • Patient steadily develops worsening of respiratory distress State 2 – Respiratory Distress Worsens • The patient rapidly deteriorates with respiratory rate increasing to 30, blood pressure falling to 80/55, heart rate increasing to 120, and SpO2 decreasing to 80% over next 4 min • Epinephrine, if administered, only provides temporizing delay in deterioration of vital signs. State 3 – Airway Secured • After airway is secured (preferably with surgical airway), SpO2 increases to 93% • If epinephrine has not been administered, patent remains hypotensive and tachycardic • If epinephrine has been administered, patient stabilizes with BP of 110/70 and HR of 96. Expected Actions – 1 CRM principles used to identify team roles and responsibilities. 2 Appropriate PPE donning prior to room entry. 3 Team leader limits number of people in room (may require expansion of scope of practice to accommodate all tasks). 4 Team leader limits equipment in room (crash cart remains outside room in “clean” area). Essential equipment carried into room. 5 Team leader articulates patient is in anaphylaxis and implements appropriate therapy, including: a High flow oxygen with precautions necessitated by COVID-19 (e.g., HFNC) b Administration of epinephrine c Considers fluid bolus d Considers diphenhydramine
6 Team leader modifies approach to securing an advanced airway for a positive COVID-19 patient. including: a Securing advanced airway early to limit aerosolization from bag/mask device. b Using a video laryngoscope instead of direct laryngoscopy. c Stating anticipation of difficult airway due to swelling d Advancing to surgical airway quicker for the CICO patient consistent with VORTEX approach [24]. 7 Communication between “dirty” team in room and “clean” team in out-of-room support roles is maintained. Debriefing Guide –The participants discuss their response with the facilitator focusing the discussion on the COVID-19 relevant aspects of the scenario. In particular, the group discusses: • donning and doffing of personal protective equipment given resource constraints and the time required to don PPE, • how many clinicians should enter the room and what roles do they assume, • clear identification of team leader, • what equipment should be brought into the room (with regards to decontamination or destruction afterward), • how to maintain communication between the “dirty” and “clean team members to solicit assistance and additional equipment, and • how treatment should be altered in the setting of COVID-19. The participants discuss their response with the facilitator focusing the discussion on the protection of clinicians during difficult airway management and the situational awareness related to the underlying anaphylaxis. In particular, the discussion could focus on: • Airway instrumentation and mask ventilation expose clinicians to virus aerosolization and the altered approach in the management of a difficult airway in a patient with contagious airborne disease. • Resource constraints on the floor in a COVID-19 pandemic regarding specialized airway equipment and practical procedural difficulties due to wearing personal protective equipment (e.g., verification of tube placement by auscultation can be hampered by PPE). • Situational awareness regarding the treatment of the underlying condition not related to the COVID-19 condition. The scenario may evoke feelings of anxiety and distress in the participants, which may come up in the discussion. Contingent on the familiarity of the participants with each other, it may be challenging to lead a discussion about concerns that touch on personal safety, professional ethics, and professional identity.Distress may be caused by • prioritizing care in the setting of insufficient hospital or ICU beds, • the delay in providing care (due to the cumbersome process of donning personal protective equipment), • the inability to assist in the patient's treatment if left as an outside (“clean”) team member • the uncertainty surrounding COVID-19 and its fatality rate, • confusing and unclear communication by leadership, • the lack of healthcare resources and absent coordinated action to confront the situation, or • the lack of personal protective equipment and resulting concerns for participants’ health or the health of family members who may be at risk due to immunocompromise, co-morbidities, simply or old age.
*Case 3: Transport of a Patient with Contagious Airborne Disease*Goal – Participants will prepare for and transport a COVID-19 positive patient from the Intensive Care Unit to the Operating Room for emergency surgery taking necessary actions to limit virus exposure to team members and others during the transport.Objectives – Participants will… 1 Employ CRM techniques including role assignments (e.g., team leader, “dirty” and “clean” team members, task assignments) and communication practices that enhance the team's shared mental model (e.g., huddle prior to room entry, closed loop communication). 2 Protect team members’ exposure by using appropriate PPE and limiting clinicians at the bedside. 3 Engage in interdisciplinary discussion with operative team to determine if procedure could be performed in patient room in order to reduce risk of exposure to bystanders along transport pathway. 4 Modify standard approach to transport for COVID-19 positive patient. including: a If not already secured, secure advanced airway in patient room to reduce risk to OR team. b Consolidate equipment to reduce “dirty” equipment being transported through facility c Discontinue any medications or fluids not essential to immediate patient needs d Replace sheets on patient bed (top and bottom) e Clean (disinfect) bed rails and exposed bed frame parts f Cover equipment to be transported to reduce virus shedding during transport g Affirm endotracheal tube security and attachment to ventilator h Transport with patient on room ventilator
5 Identify team member roles for transport, including: a Which team members remain “dirty” b Standby team member in full PPE, but remains “clean” (no patient contact unless needed) c Security or other staff ahead of transport to clear hallways, close room doors, and open upcoming hallway doors d Security or transport contacted to obtain elevator access e Environmental health services on scene with person to follow transport team and clean pathway floors after passing Equipment and Supplies – • COVID-19 specific Personal Protective Equipment (e.g., mask, face shield, gown, gloves and/or PAPR) • Ventilator • IV pumps (2) on separate IV poles – Fluids and medications in Simulator and Environment setup • Clean sheets (top and bottom) • Clear plastic bags (large) • Transport monitor • Stethoscope Simulator and Environment – • Manikin capable of being ventilated by ventilator • Manikin on ICU bed • Wearing hospital gown • Vital signs (EKG, NIBP, EtCo2, and SpO2) on display in room • IVs in place (3) ○ Flolan (epoprostenol sodium) – Connected to ventilator circuit for aerosolization ○ Fentanyl ○ Normal saline at KVO rate • Intubated, with tube secured and connected to ventilator • Foley catheter placed with collection bag In Scenario Actor – • Faculty member in role of bedside clinician to supply relevant patient information. Faculty member is in full PPE appropriate for a COVID-19 patient. Scenario Duration – • Briefing – 3 min • Simulation – 15 min • Debriefing – 12 min Briefing –“Your team has been called to the ICU to transport a COVID-19 positive patient to the Operating Room for an emergent hemicraniectomy. The Operating Room is two floors below the patient's unit in the North Wing. The ICU is in the South Wing.”Simulation Progression –In scenario actor meets team in hallway outside patient room, stating… • “I am the nurse assigned to this patient. We have a 42-year-old male patient who was in a motor vehicle collision. He needs transport to the Operating Room for a hemicraniectomy. He is intubated and on the ventilator. He currently has two medications running – Flolan and fentanyl.” Upon questioning, the nurse supplies the following… • There is no remarkable medical, surgical, or social history and patient has no known medication allergies. • He is a computer programmer. • His-drug screen came back negative. • He was involved in a single vehicle crash into a tree. Unrestrained driver. Closed head injury. • Colleagues reported he had flu-like symptoms with fever over past few days. • He has no recent travel history and no known contacts with COVID-19 confirmed individuals. Patient Presentation – • Patient is intubated and on ventilator, connected to ICU monitor, with medication IV pumps running. He is unresponsive.
Physical examination – • Patient weight is 79 KG. Height is 179 cm. • Lung sounds indicate rales • Bruising to right forehead Vital Signs • Heart Rate – 78 • Respiratory Rate – 16 • Blood Pressure – 117/83 • SpO2 – 98% on FiO2 of 50%, PEEP at 14, TV of 500, and RR at 14 with SIMV • Temperature – 38.9 C Initial State (3 min) • Patent remains stable throughout scenario, provided no incident during transport. • Will require transport from room to another location or, as alternative, return to origination room after transport through hallways. Expected Actions – 1 CRM principles used to identify team roles and responsibilities. 2 Appropriate PPE donning prior to room entry. 3 Team leader limits number of people in room (may require expansion of scope of practice to accommodate all tasks). 4 Team leader limits equipment in room 5 Team leader considers interdisciplinary discussion with surgical team to determine if procedure can be done in the ICU. 6 Team leader coordinates team members to implement preparations for transport, including: a Consolidate equipment to reduce “dirty” equipment being transported through facility i Only transport one IV pump b Discontinue any medications or fluids not essential to immediate patient needs i Flolan needs to continue ii Fentanyl and NS can be stopped for transport c Replace sheets on patient bed (top and bottom) d Clean (disinfect) bed rails and exposed bed frame parts e Cover equipment to be transported to reduce virus shedding during transport i Use clear large plastic bag – screens still visible and device touch screen interfaces still work ii Affirm endotracheal tube security and attachment to ventilator iii Transport with patient on room ventilator 7 Team leader coordinates role for transport process, including: a Which team members remain “dirty” b Standby team member in full PPE, but remains “clean” (no patient contact unless needed, holds gloves in hands as reminder they are “clean” until needed.) c Security or other staff ahead of transport to clear hallways, close room doors, and open upcoming hallway doors d Security or transport contacted to obtain elevator access e Environmental health services on scene with person to follow transport team and clean pathway floors after passing 8 Communication between “dirty” team in room and “clean” team in out-of-room support roles is maintained. Debriefing Guide –Team coordination, communication with hospital entities, and adherence to protocol to contain viral spread are the core elements of this case discussion. The team reflects on its own performance then facilitators comment with observations or protocol breaches. The focus of the ensuing discussion could be on • Pro-and cons of performing critical procedures in the patient's room versus another location, with special consideration of negative or positive pressure setup in airborne isolation rooms versus the normal operating room. • Optimal preparation for transport by limiting infusion pumps, covering accompanying equipment to reduce contamination. • Intubation prior to transport versus intubation to the operating room with a view to reduce exposure of OR personnel and hallway bystanders by avoiding bag mask ventilation outside the negative pressure environment.
*Case 4: Tele-instruction for Remote Procedural Guidance and Supervision*Goal – Remote team leader will provide instruction and coaching to team members using a two-way audio/visual link (telehealth) to instruct bedside participant how to perform chest tube placement in a patient with a tension pneumothorax that has been temporized with needle thoracotomy.This scenario requires the “Hot Seat” participant in the role of the remote team leader to be experienced in the procedure and the bedside clinician to be relatively inexperienced or not current in practice of the procedure. For this reason, participant roles were specially designated.Note – This section details the telehealth interaction between the remote team leader and the bedside clinician(s). This section was preceded by the bedside team assessing and intervening with a patient presenting with possible tension pneumothorax. The “Hot Seat” participant was the remote team leader. The first part of this scenario was conducted to provide another training opportunity for participants to cover key objectives presented in other simulation scenarios regarding team management in COVID-19 patients.Objectives (specific to telehealth interaction for chest tube placement) – 1 Remote team leader engages in positive communication practices using techniques to enhance success, including: a Speaking in clear, calm voice b Providing specific step-by-step instructions c Providing encouragement and reassurance to bedside clinician 2 Remote team leader enhances the video environment by directing movement of the camera, patient, or provider to obtain clear view of the procedure. 3 Remote team leader provides opportunity for bedside clinician to ask questions and provide information essential to the case. Equipment and Supplies – • COVID-19 specific Personal Protective Equipment for bedside clinician(s) (e.g., mask, face shield, gown, gloves and/or PAPR) • Two-way audio/visual communication (telehealth system or other system such as Zoom or Go to Meeting) • Chest tube tray or equivalent equipment and supplies • Chest tubes, assorted sizes • Water seal chest drainage unit • Suction Simulator and Environment – • Chest tube simulator or manikin capable of inserting chest tube (with incision) • Work surface for chest tube simulator or height adjustable stretcher • Will require two rooms (simulation room and remote telehealth room) In Scenario Actor – • None required for telehealth section of scenario. Scenario Duration – • Briefing – 3 min • Simulation – 12 min • Debriefing – 15 min Briefing (to remote team leader) –“You have been asked to do a teleconsultation with a physician at another hospital who is managing a COVID-19 positive patient who now requires a chest tube after successful needle thoracotomy in the right chest. The provider has done the procedure before but is out of practice and requesting assistance. The patient was in the ICU and had been diagnosed with ARDS related to COVID-19 diagnosis. A central line had been placed immediately before patient deterioration due to tension pneumothorax.”Simulation Progression –Patient Presentation – • Patient is now stable after needle thoracotomy • 60-year-old male truck driver • There is no remarkable medical, surgical, or social history and patient has no known medication allergies. • Only medications are over the counter pain relievers Physical examination – • Patient weight is 88 KG. Height is 173 cm • Lung sounds indicate rales present on right but slightly diminished, clearly audible on the left • Central line in right subclavian
Vital Signs • Heart Rate – 88 • Respiratory Rate – 20 • Blood Pressure – 117/73 • SpO2 – 94% on 4 lpm nasal cannula • Temperature – 38.8 C Initial State • Patent remains stable throughout scenario Expected Actions – 1 Remote team leader introduces self and asks for summary of situation (SBAR). 2 Remote team leader assesses situation to determine urgency. 3 Remote team leader optimizes field of view by either moving camera location or moving patient into field of view. 4 Remote team leader guides bedside clinician(s) through procedure while using positive communication tactics, including: a Speaking in clear, calm voice b Providing specific step-by-step instructions c Providing encouragement and reassurance to bedside clinician 5 Two-way communication maintained throughout scenario. Debriefing Guide –The remote team leader serving as the telehealth consultant and the bedside clinician discuss their team cooperation during the chest tube insertion. Other participating or observing participants are also asked to contribute.Besides practice in a rare but potentially lifesaving procedure, the central theme is communication and supervision via remote telecommunication. In particular, the discussion could focus on • Developing a shared mental modeling prior to engaging in a critical procedure, (e.g. with a dry run or procedure stepwise rehearsal if time permitted), • Optimizing communication and visual contact prior to the procedure (e.g. align camera and patient, moving the more mobile of the two), • The manner and style of communication between the two key participants to determine what enhanced communication and what may have been a barrier. This scenario focused on a remote telehealth situation. The same techniques could be employed locally when the clinician at the patient's bedside needs assistance and due to limiting the number of people in the COVID-19 patient room or inability of the supervising physician to enter the room (e.g., on home quarantine, underlying health issue that places the senior provider at high risk).

**Abbreviations:** CICO: can't intubate, can't oxygenate, EKG: Electrocardiogram, NIBP: noninvasive blood pressure, EtCo2: end-tidal carbon dioxide SpO2: peripheral oxygen saturation, IV: intravenous, KVO: keep vein open.

## 3 Experimental design, materials and methods

### Simulation context, setup, and debriefing

When we conducted our simulations in March 2020, hospitals resources were already stretched in New York City. Penn State teams had started to develop protocols to guide clinicians in expected COVID-19 scenarios, based on the sparse medical literature available at that time. However, no one on our teams had gained any personal experience managing patients with COVID-19[8,9,11], as no COVID-19 patients had been yet admitted at our institution, the Penn State Health Milton S. Hershey Medical Center (MSHMC), in Hershey, Central Pennsylvania. We converted a previously scheduled MOCA (Maintenance of Certification in Anesthesiology) simulation course [6,7,19] in our American Society of Anesthesiologists Endorsed simulation program to train internal clinicians in scenarios related to COVID-19. The simulations were hence geared towards experienced anesthesiology, perioperative and critical care physicians. Several authors (MHA, ES, DLR, VC and AD) designed four cases for seven participating physician anesthesiologists, who would each be in the “hot seat”, (the critical central active role of the scenario), at least once. [20] All participants and course instructors were employed by the Medical Center and contributed as co-authors to this manuscript.

### Case scenarios simulated anticipated COVID-19 clinical encounters

The sequence of our simulations was as follows. All participants initially participated in three skills practice sessions expected to be relevant to patient care: 1) donning and doffing of PPE; 2) emergency surgical airway; and 3) intraosseous access[9,21]. This was followed by case scenarios beginning with a code blue/cardiac arrest in a patient with a history consistent with COVID-19 infection, but without confirmatory or exclusionary test results available. One participant was given the role of the team leader and the rest were expected to act as the response team[20]. This case was debriefed first on the teamwork and communication aspects of the code response along the lines of usual CRM (Crisis Resource Management) debriefing [22,23] and then the entire group discussed aspects of the case specifically relevant to changes in practice specific to managing a cardiac arrest patient in the context of possible COVID-19 infection[12]. The same case was repeated with a different team leader and the group practiced the protocol that had been designed for COVID-19 patients for management of cardiac arrest. The debriefing that followed focused primarily on how the protocol worked and what needed to be changed or refined in the protocol.

Next, two scenarios for known COVID-19 patients were run simultaneously for two different teams. One case required the team to transport an intubated patient to the operating room, and the other case was a difficult emergency airway where the patient cannot be intubated or oxygenated requiring an emergency surgical airway[9,24,25]. These cases were debriefed for CRM concepts[22,23] and then new protocols for patient transport and emergency airway management were discussed and suggestions made for revisions. The final case was a tension pneumothorax in a COVID-19 patient that is relieved by needle decompression. The participant in the room with only minimal experience in placing a chest tube is provided with a telemedicine link to an expert (the hotseat participant) who must guide them through the procedure remotely[26]. The CRM debriefing for this case was followed by a discussion of available telehealth resources at our institution, practical aspects of how to access those, and communication behaviors that facilitated effective remote telepresence guidance of the procedure.

## Ethics statement

All participants and course instructors volunteered to participate in this educational project and contributed as co-authors to this manuscript.

## Declaration of Competing Interest

No author has any competing interest.

## Appendix Supplementary materials

Image, application 1
